# Supplementary material for: An oligomeric state‐dependent switch in the ER enzyme FICD regulates AMPylation and deAMPylation of BiP
Source: EMBO J. 2019 Sep 18;38(21):e102177. doi: 10.15252/embj.2019102177 (PMC6826200; doi:10.15252/embj.2019102177)
Supplement: Supplementary file 1 — Appendix [file EMBJ-38-e102177-s001.pdf]

APPENDIX

An oligomeric state-dependent switch in the ER enzyme FICD regulates AMPylation and deAMPylation of BiP

Luke A. Perera, Claudia Rato, Yahui Yan, Lisa Neidhardt, Stephen H. McLaughlin, Randy J. Read, Steffen Preissler, David Ron.

Contents:

Appendix Figure S1
Appendix Figure S2

Appendix Figure S1

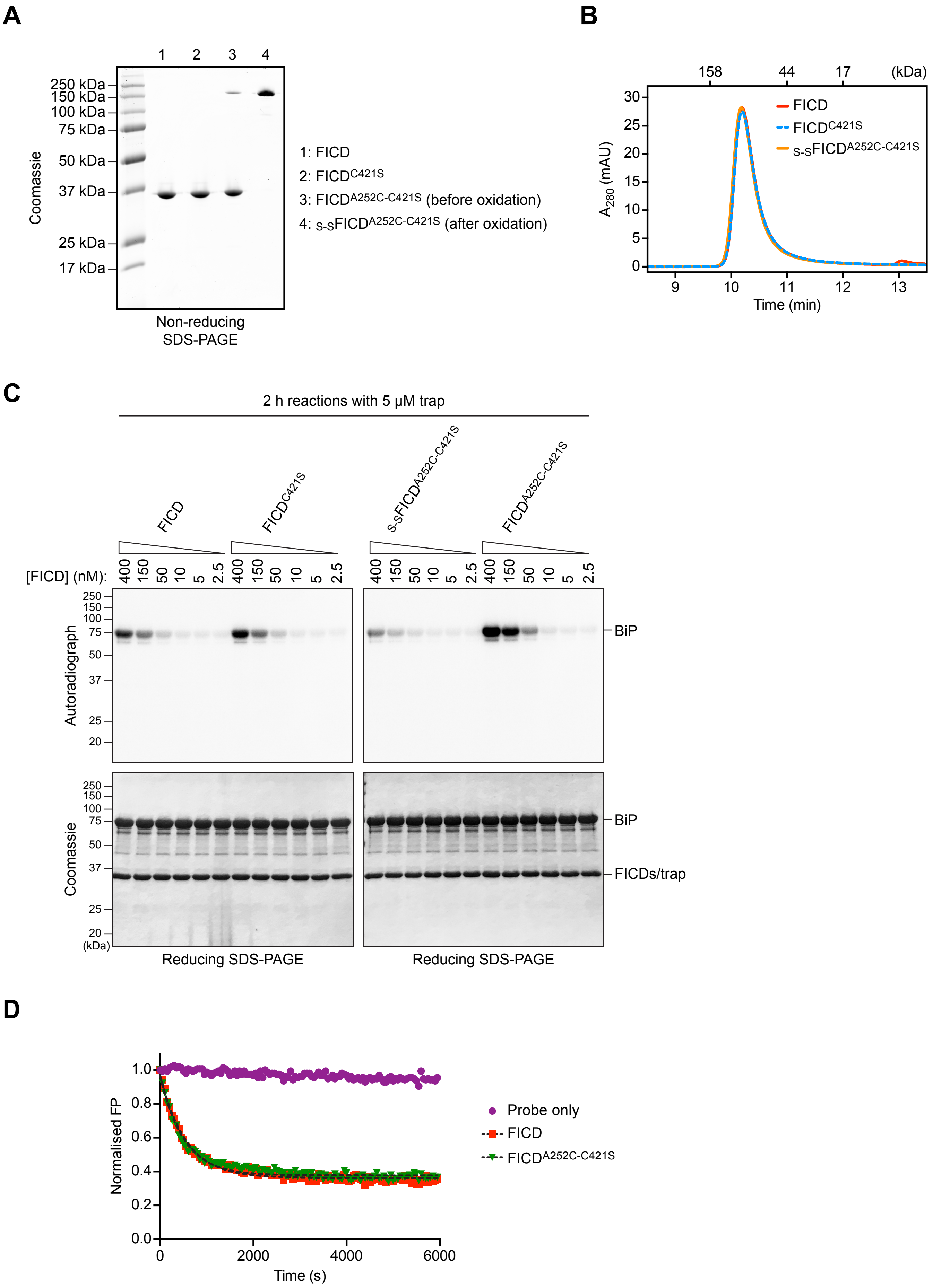

Appendix Figure S1 - Non-disulphide-linked FICD$^{A252C-C421S}$ shows enhanced AMPylation activity.

A Coomassie-stained, non-reducing SDS-PAGE gel of the indicated FICD proteins.

B Size exclusion chromatography (SEC) elution profiles of FICD proteins injected at a concentration of 20 µM. Protein absorbance at 280 nm is plotted against elution time. The elution times of protein standards are indicated as a reference. Note that wild-type FICD, FICD^{C421S}, and oxidised $_{S-S}$FICD$^{A252C-C421S}$ co-elute as dimers. See Fig EV1D-E.

C Radioactive in vitro AMPylation reactions were performed as in the right-hand side panel of Fig 3A, that is with the indicated FICD proteins under non-reducing conditions in presence of covalently linked $_{S-S}$FICD$^{A252C-H363A-C421S}$ dimers (trap). Note that the accumulation of modified BiP correlates with the FICD concentration. Less modified BiP was produced by covalently-linked, oxidised $_{S-S}$FICD$^{A252C-C421S}$ dimers, whereas more AMPylated BiP was generated in reactions containing non-oxidised FICD$^{A252C-C421S}$. The trap, present at 5 µM, co-migrates with the indicated FICD enzyme and dominates the signal in the Coomassie stained gel (FICD/trap).

D Time-dependent in vitro deAMPylation of fluorescent BiP^{V461F}-AMP^{FAM} by the indicated FICD proteins (at 7.5 µM) assayed by fluorescence polarisation (as in Fig 2A). A representative experiment (data points and fit curves) is shown and deAMPylation rates are presented in Fig EV2A. Note that non-oxidised FICD$^{A252C-C421S}$ has very similar deAMPylation kinetics to the wild-type protein. This contrasts with the oxidised form which displays a slight increase in deAMPylation rate (Figs 3D and EV2A).

Appendix Figure S2

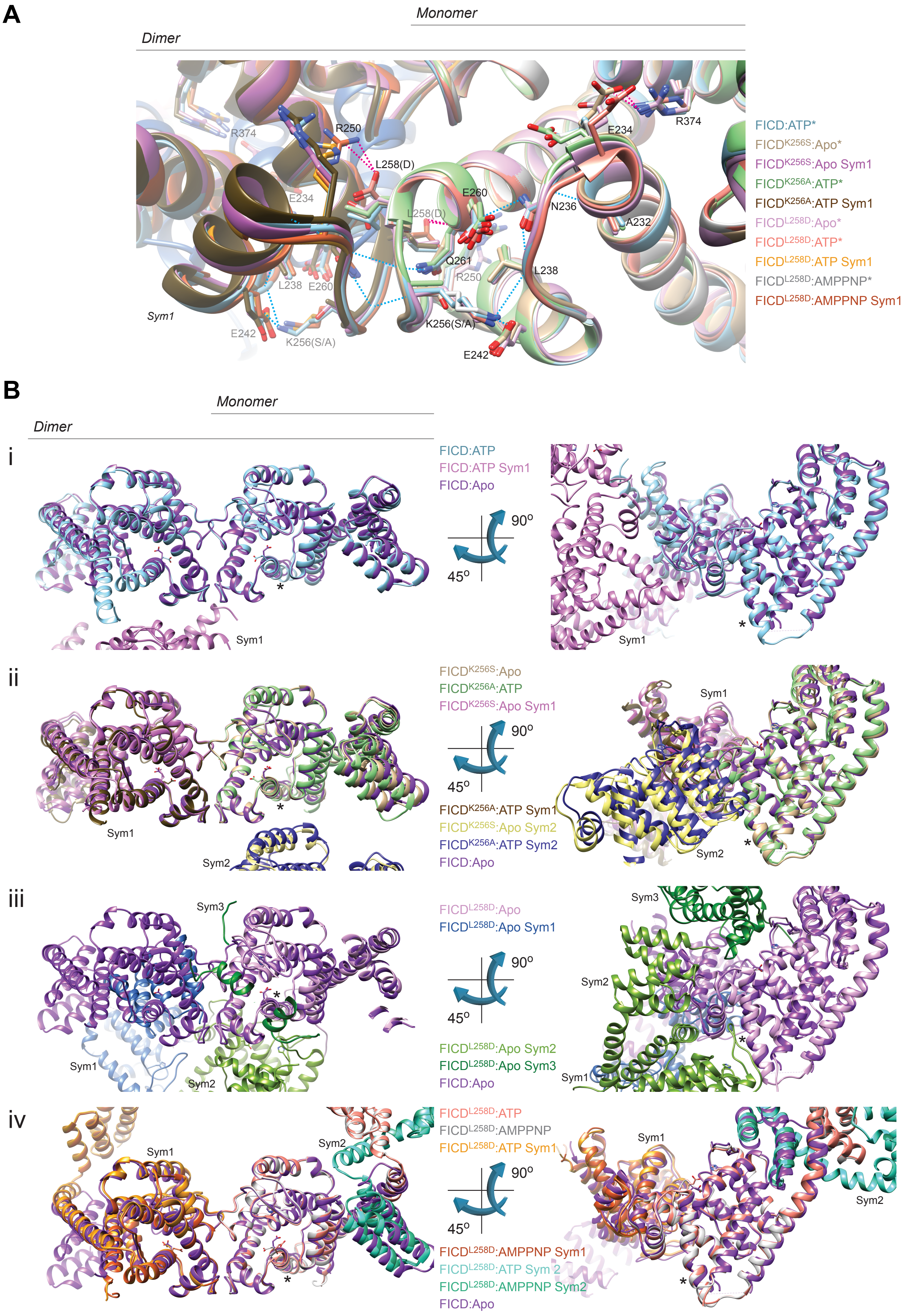

Appendix Figure S2 - FICD crystallographic packing and dimer interface.

A The hydrogen bonding network connecting the dimer interface and enzyme active site is maintained in the crystal structures of monomeric and Lys256 mutant FICDs. An alignment of the hydrogen bond network linking the dimer interface to Glu234 in the indicated structures is displayed (in the same view as Fig 4A). H-bonds are shown in blue dashed lines. Where indicated, single molecules from the asymmetric unit (*) are displayed with their respective symmetry mates (Sym1). Note, the side-chains of Asp258 and (Sym1)Arg250 of the monomeric FICD^{L258D} (cocrystallised with nucleotide) form a crystallographically induced inter-molecular H-bond (magenta dashed line). The salt-bridges between the Glu234 and the Fic motif Arg374 (magenta dashed lines) in the FICD^{L258D}:Apo and FICD^{K256S}:Apo structures, observed in other inhibitory glutamate-containing Fic crystal structures, are also shown.

B Dimer interface contacts are imposed crystallographically, and crystal packing around the a_{inh} is similar in all FICD structures. FICDs with similar crystal packings are grouped into panels *(i-iv)*. The inhibitory alpha helix (α_{inh}) is denoted with an asterisk (*) and Glu234s are shown as sticks. The wild-type dimeric FICD:Apo structure (FICD:Apo; PDB: 4U0U) is provided in all panels for reference. Where a single FICD molecule constituted the asymmetric unit, symmetry mates within 4 Å of its dimer interface (Sym1) or 4 Å of its inhibitory helix region (Sym2/3) are also displayed. Note that crystals of the Lys256 mutants *(ii)* contain a single molecule in their asymmetric unit but are packed as dimers, crystallographically reconstituting the dimeric biological unit. The asymmetric unit of FICD^{L258D} bound to ATP (or an ATP analogue) *(iv)* contains a single molecule and thus corresponds to the biological unit of this monomeric protein. However, packing against its symmetry mates (Sym1), crystallographically reconstitutes a dimer interface that is highly similar, but not identical, to that observed in the wild-type protein (see Asp258 and (Sym1)Arg250 in S2A, above). Sym2 in *(iv)* serves to highlight the replacement of the flipped out TPR domain with the flipped out TPR domain from a symmetry mate. In *(iv)* there are no crystal contacts in the vicinity of the α_{inh}.
