# Supplementary material for: An oligomeric state‐dependent switch in the ER enzyme FICD regulates AMPylation and deAMPylation of BiP
Source: EMBO J. 2019 Sep 18;38(21):e102177. doi: 10.15252/embj.2019102177 (PMC6826200; doi:10.15252/embj.2019102177)
Supplement: Supplementary file 2 — Expanded View Figures PDF [file EMBJ-38-e102177-s002.pdf]

Expanded View Figures

Figure EV1. Low-level expression facilitates AMPylation *in vivo* and FICD mutations are able to disrupt the tight dimer formed in solution.

A Schematic representation of the domain organisation of FICD and the shorter protein fragment used for *in vitro* experiments. The transmembrane domain (blue), the TPR domain (orange), the α-helical linker (green), the Fic domain (purple) and the core Fic domain (deep purple) including the active site motif are indicated.

B, C Characterisation of CHO-K1 $FICD^{-/-}$ UPR reporter clones stably expressing wild-type (wt) FICD. (B) Flow cytometry analysis of CHO-K1 $FICD^{-/-}$ UPR reporter clones stably expressing mCherry and FICD. Clones were selected based on mCherry signal, assuming a direct correlation with FICD expression levels. (C) Immunoblot of endogenous BiP from CHO-K1 $FICD^{-/-}$ clones shown in (B) exposed to cycloheximide as in Fig 1A. Note that only clone 10, with an intermediate mCherry signal, showed detectable accumulation of AMPylated BiP.

D, E Size-exclusion chromatography (SEC) analysis of wild-type and mutant FICD proteins. (D) SEC elution profiles with FICD proteins at the indicated concentrations. Black dots mark the position of the elution peaks. Dotted lines mark the approximate elution peak times for dimeric (10.2 min) and monomeric (11.4 min) FICD, respectively. Absorbance at 280 nm ($A_{280\ nm}$) is plotted in units of milli-absorbance units (mAU). (E) Plot of the elution peak times from (D) as a function of protein concentration. With the exception of $FICD^{G299S}$ (*a mutation that shifts the elution time relative to the monomer), best-fit monomer-dimer association curves are shown with the top plateau constrained to the monomer elution time (11.4 min). Approximate dimerisation K_ds were derived and are shown in the figure key for the different partially monomerising mutants (with 95% confidence intervals). Note that $FICD^{L258D}$ eluted as a monomer and wild-type FICD principally as a dimer at all concentrations tested (0.2–50 μM). Conversely, $FICD^{G299S}$ and non-oxidised $FICD^{A252C-C421S}$ formed much weaker dimers. As in (D), the monomer and dimer elution times are represented by dotted (horizontal) lines.

F, G Analysis of FICD by analytical ultracentrifugation. Overlays of *c*(*s*) distributions of (F) wild-type FICD and (G) $FICD^{L258D}$ are shown in units of experimental *s*-values. A signal-weighted isotherm for the wild-type protein (Fig 1E) was generated from integration of the titration series distributions.

© 2019 The Authors

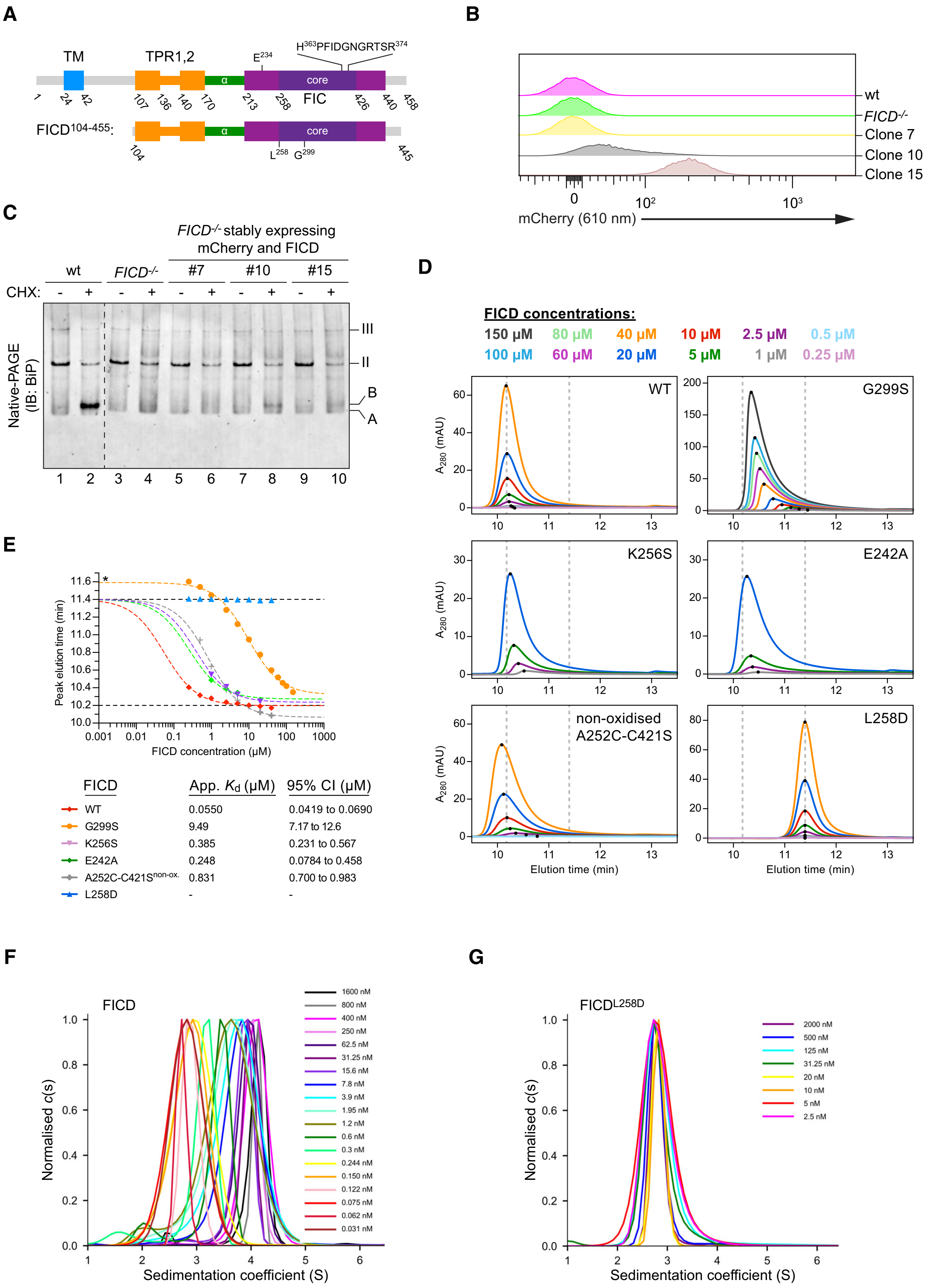

Figure EV1.

 © 2019 The Authors

Figure EV2. Monomerisation inhibits deAMPylation and markedly stimulates FICD AMPylation activity.

A Summary of deAMPylation rates of wild-type and mutant FICD proteins. Shown are deAMPylation rates of BiP^{V461F}-AMP^{FAM} by the indicated FICD proteins (at 0.75 μM or 7.5 μM) as detected by a change in fluorescence polarisation. Mean decay rate constant values ± SD from normalised raw data fitted to mono-exponential decay functions of at least four independent measurements are presented.

B, C The effect of FICD overexpression on a UPR reporter. (B) Flow cytometry analysis of wild-type (wt) and $FICD^{-/-}$ CHO-K1 *CHOP::GFP* UPR reporter cells transfected with plasmids encoding wild-type or the indicated FICD derivatives and a mCherry transfection marker. Shown are the median values ± SD of the GFP fluorescence signal of mCherry-positive cells from three independent experiments (fold change relative to wild-type cells transfected with a plasmid encoding mCherry alone). Note that only Glu234Gly-containing, deAMPylation-deficient FICDs activate the reporter. (C) Flow cytometry raw data of a representative experiment.

D AMP production by FICD dimer interface or relay mutants is BiP dependent. AMP production in the presence of [α-^{32}P]-ATP was measured by TLC and autoradiography (as in Fig 2B). Plotted below are mean AMP values ± SD from three independent experiments.

E–G Characterisation of covalently linked $_{S\text{-}S}FICD^{A252C\text{-}H363A\text{-}C421S}$ dimers—a trap for BiP-AMP. (E) Coomassie-stained, SDS–PAGE gel of the indicated FICD proteins. (F) Size-exclusion chromatography elution profiles of wild-type FICD and covalently linked $_{S\text{-}S}FICD^{A252C\text{-}H363A\text{-}C421S}$ (trap) dimers at 20 μM, as in Fig 1D. Note that the oxidised trap elutes, like the wild-type FICD, as a dimer. (G) BioLayer interferometry (BLI)-derived association and dissociation traces of the indicated FICD proteins (in solution) from immobilised AMPylated (BiP-AMP) or unmodified BiP. The trap ($_{S\text{-}S}FICD^{A252C\text{-}H363A\text{-}C421S}$) and $FICD^{H363A}$ had indistinguishable tight interaction with BiP-AMP (with low off rates). The interaction of BiP-AMP with monomeric $FICD^{L258D\text{-}H363A}$ was more transient. The interaction between these FICD variants and unmodified BiP was further diminished.

H Sequestration of AMPylated BiP by trap FICD analysed by SEC. Elution profiles of *in vitro* AMPylation reactions containing the indicated components in the presence or absence of covalently linked $_{S\text{-}S}FICD^{A252C\text{-}H363A\text{-}C421S}$ (trap) dimers to sequester the AMPylated BiP product. Note that the trap forms a stable complex with BiP when AMPylated by monomeric $FICD^{L258D}$. An early eluting species, representing a stable complex between modified BiP and trap, only occurs in the reaction containing AMPylation-active, monomeric $FICD^{L258D}$ and ATP (bottom right panel, pink trace). Here, BiP-mediated ATP hydrolysis and substrate interactions were discouraged by use of a $BiP^{T229A\text{-}V461F}$ double mutant. mAU, milli-absorbance units.

Source data are available online for this figure.

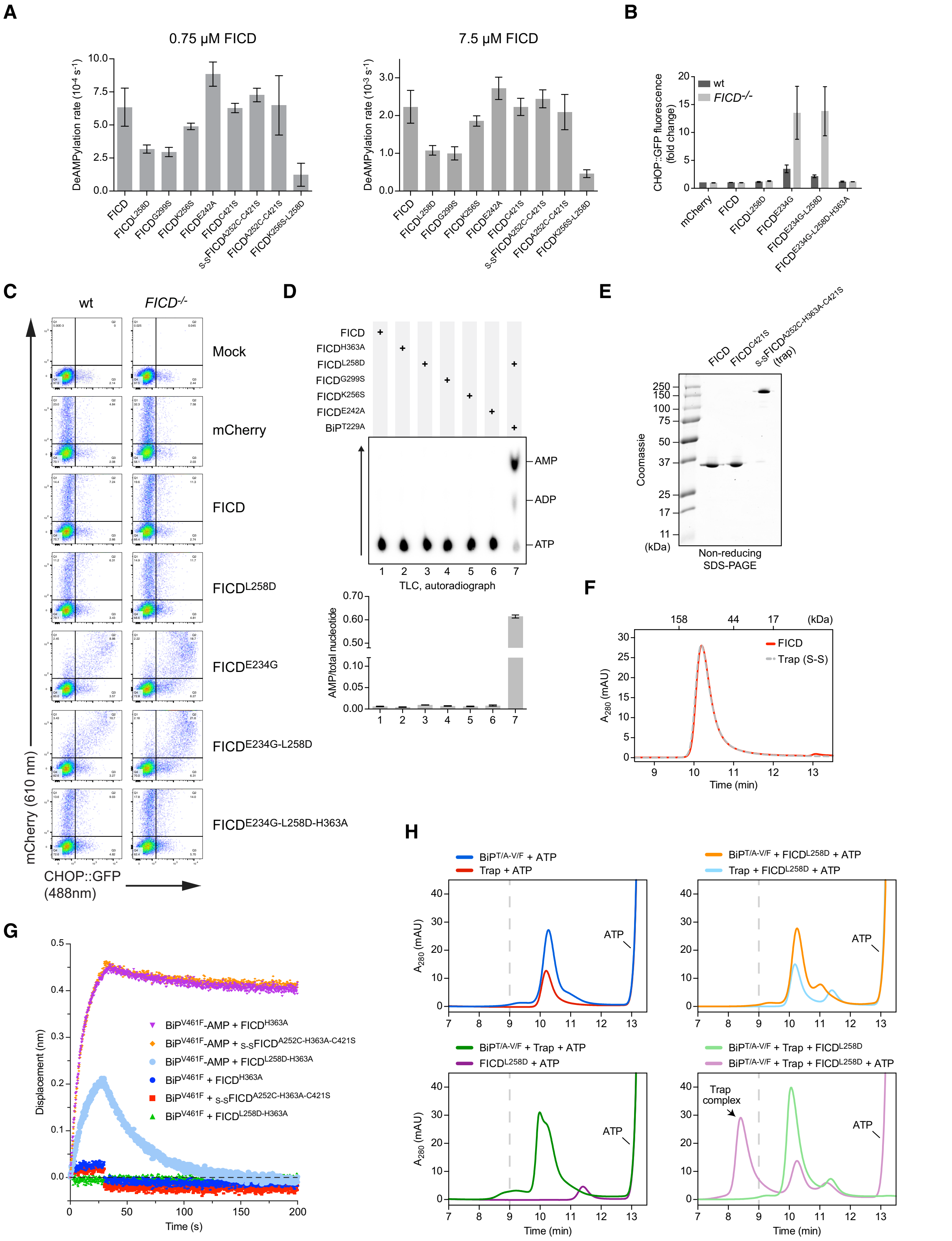

Figure EV2.

© 2019 The Authors

Figure EV3. FICD dimer relay mutants produce a pool of AMPylated BiP *in vitro*, and FICD AMPylation activity correlates with increased flexibility.

A Radioactive *in vitro* AMPylation reactions with the indicated FICD proteins at the indicated concentrations, [α-^{32}P]-ATP, and BiP$^{T229A-V461F}$ were analysed by SDS–PAGE. The radioactive signals were detected by autoradiography and proteins were visualised by Coomassie staining. Note the enhanced production of AMPylated BiP in the presence of dimer relay mutants, FICD^{K256S} and FICD^{E242A}, relative to the wild-type protein and a further increase in the production of AMPylated BiP by the monomeric FICD$^{K256S-L258D}$ double mutant relative to monomeric FICD^{L258D}. Also note the auto-AMPylation signals of the monomeric FICDs detectable at high enzyme concentration.

B, C *In vitro* deAMPylation of fluorescent BiP^{V461F}-AMP^{FAM} by the indicated FICD proteins (at 7.5 μM) measured by fluorescence polarisation. A representative experiment (data points and fit curves) is shown and rates are presented in Fig EV2A. Note the impaired deAMPylation activity of the monomeric FICD$^{K256S-L258D}$ double mutant in (C).

D DSF T_m analysis of wild-type (wt) and mutant FICD proteins in absence (Apo) or presence of ATP or ADP. Nucleotide concentrations in mM are given in parentheses. Non-oxidised and oxidised forms of FICD$^{A252C-C421S}$ were assayed in buffer lacking reducing agent (which did not affect the T_m of wild-type FICD; see source data). Shown are the mean T_m values ± SD from three independent experiments. Note that FICD^{K256A} is more stable than FICD^{K256S} but less than wild-type FICD. Furthermore, the stabilities of oxidised and non-oxidised FICD$^{C421S-A252C}$ relative to the wild-type correlate inversely with their AMPylation activities (Fig 3B). For the wild-type FICD, FICD^{E242A}, FICD^{G299S}, FICD^{L258D} and FICD$^{K256S-L258D}$, in the apo state, the same data are presented in Fig 4E.

E Plot of the increase in FICD melting temperature (ΔT_m) against ATP concentration as measured by DSF (derived from Fig 4F). Note the similarity in the $K_{1/2}$s of ATP-induced T_m increase (annotated) between FICD^{L258D} (mFICD) and the wild-type dimer (dFICD). Shown are mean ΔT_m values ± SD of three independent experiments with the best-fit lines for a one-site binding model.

Source data are available online for this figure.

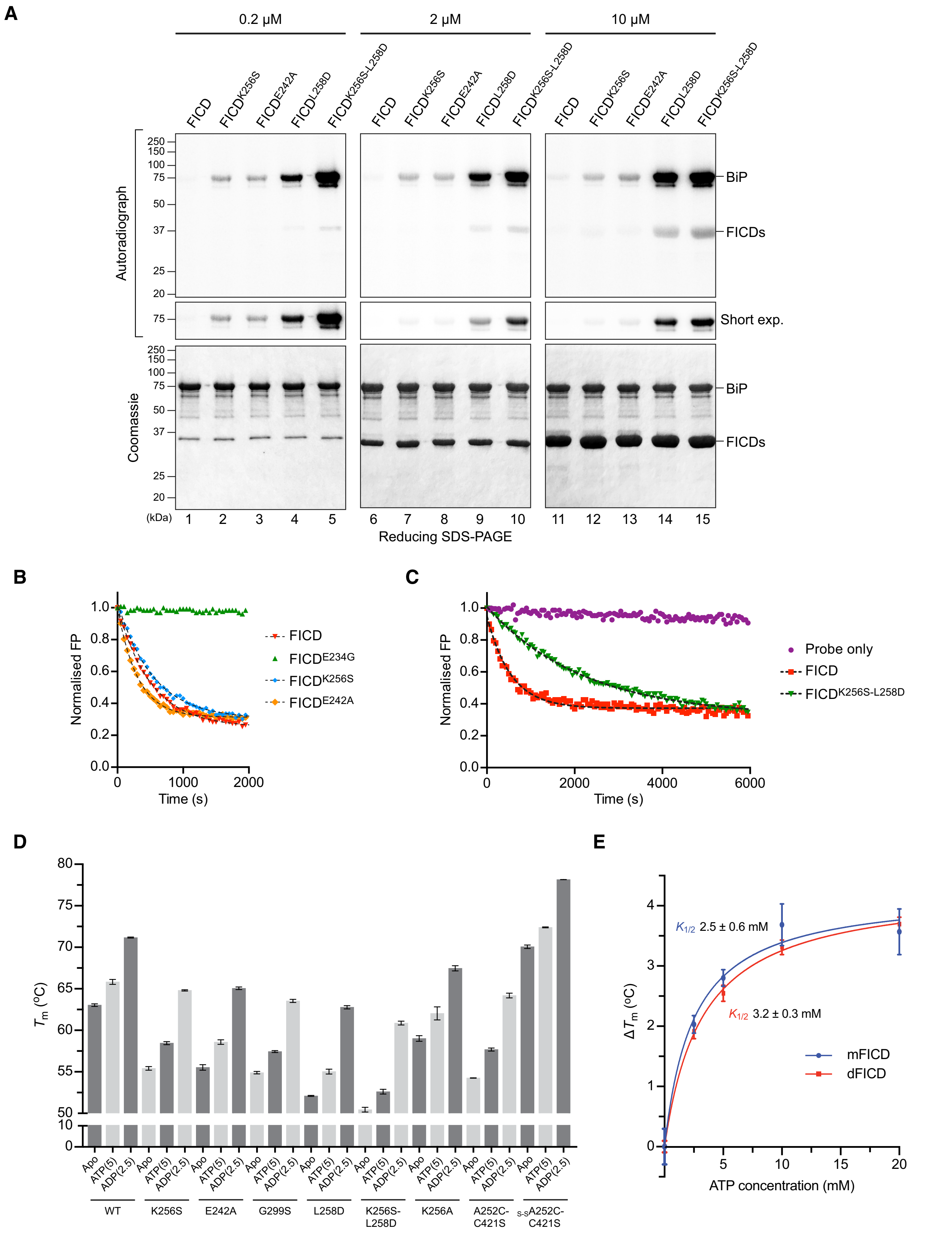

Figure EV3.

 © 2019 The Authors

Figure EV4. Monomerisation allows ATP to bind to FICD in a mode conducive to BiP AMPylation.

A Mutation of the dimer relay residue Lys256 does not result in large conformational changes in FICD. Shown is the alignment (residues 213–407) of the molecules in the asymmetric unit. Structures are coloured as indicated. Glu234, ATP and Mg (where applicable) are shown as sticks. The inhibitory alpha helix (α_{inh}) and gross domain architecture is annotated. The FICD:Apo structure is from PDB: 4U04.

B Electron density of both MgAMPPNP and the inhibitory Glu234, from monomeric FICD^{L258D} co-crystallised with MgAMPPNP. Unbiased polder (OMIT) maps are shown in blue and purple meshes, contoured at 3.0 and 5.0σ, respectively. All residues and water molecules interacting with MgAMPPNP are shown as sticks and coloured by heteroatom. Mg^{2+} coordination complex pseudo-bonds are show in purple dashed lines.

C Unlike wild-type FICD, monomeric FICD^{L258D} binds ATP and ATP analogues in an AMPylation-competent conformation. The indicated structures and distances are shown as in Fig 5C, with ATP interacting residues shown as sticks and annotated. The position of the α-phosphate relative to Val316 in the FICD:ATP structure (see distances in right-hand side panel) would preclude in-line nucleophilic attack (see D, E). The inset is a blow-up displaying distances (i–iv) between the γ-phosphates and Glu234 residues. A potentially significant difference in the Glu234 position between the FICD^{L258D}:MgAMPPNP and FICD:ATP structures is apparent: hypothetical distance (ii) (2.68 Å, between Glu234 of FICD:ATP and AMPPNP γ-phosphate of FICD^{L258D}) is less favourable than the observed distance (iii) (2.94 Å, between the AMPPNP γ-phosphate and Glu234 of FICD^{L258D}). Note, His363 of FICD:ATP is in a non-optimal flip state to facilitate general base catalysis (see Fig 5B).

D (i) The mode of ATP binding in wild-type dimeric FICD sterically occludes the nucleophilic attack required for AMPylation. Shown are semi-opaque 3 Å centroids centred on Pα and Val316 (Cγ1). The putative BiP Thr518 nucleophile (depicted by the cross) is positioned in-line with the scissile phosphoanhydride (parallel to the plane of the paper) and 3 Å from Pα. This nucleophile position lies within the Val316 centroid (indicating a steric clash). For clarity, the FICD:ATP structure is overlaid with a thin slice of the FICD:ATP structure in the plane of the Pα-O3α bond. (ii) In the monomeric AMPylation-competent FICD^{L258D}:ATP structure, the nucleophile lies outside the Val316 centroid in proximity to His363 (the general base).

E The ATP α-phosphates of monomer or dimer relay mutants are in the same position as that competently bound to the AMPylation unrestrained dimeric FICD^{E234G}. Shown are all AMPylation-competent MgATP structures overlaid as in (C) and Fig 5C. The dimeric FICD^{E234G}:MgATP (dark blue, PDB: 4U07) is also included as a reference for an active AMPylating enzyme.

© 2019 The Authors

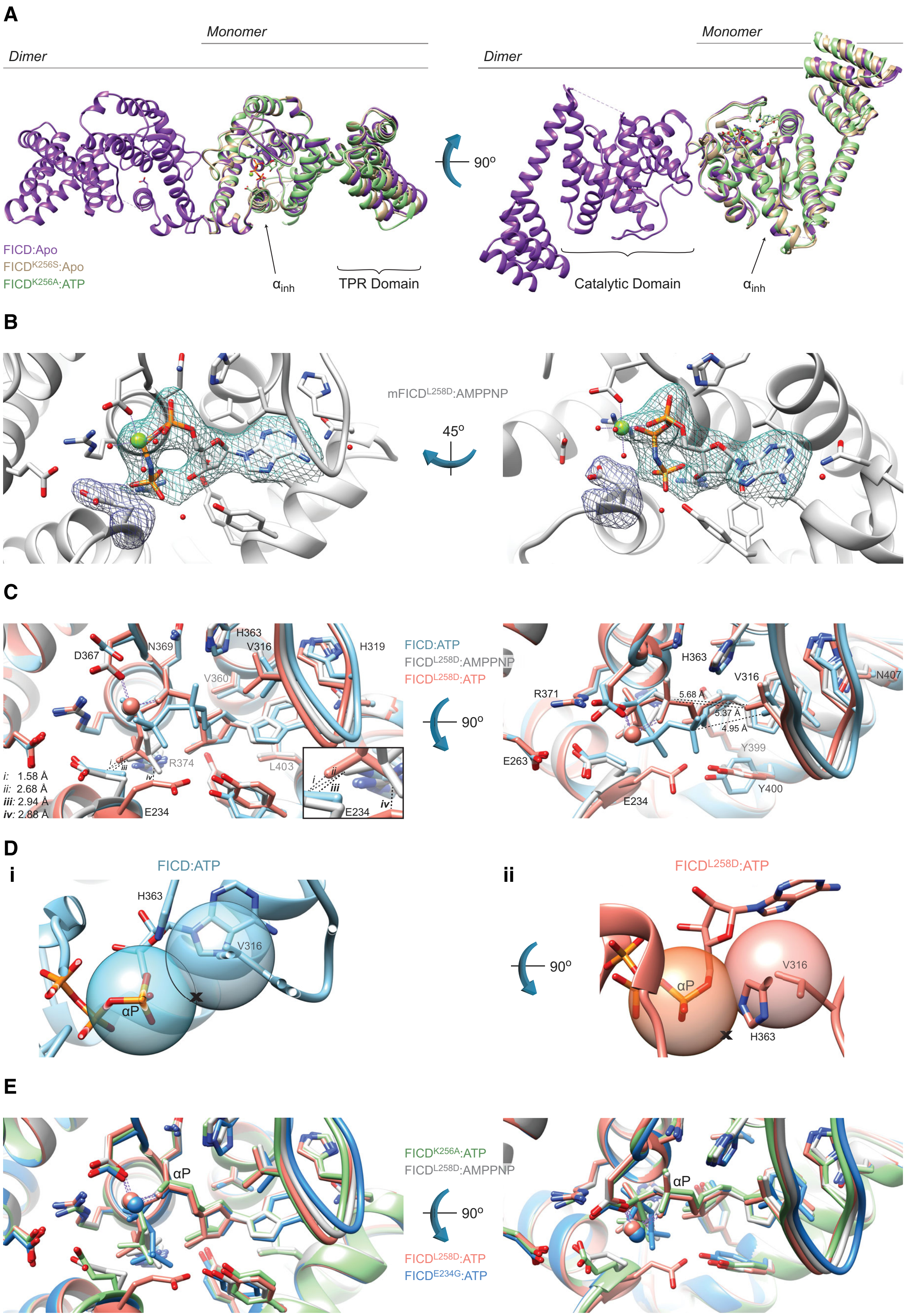

Figure EV4.

 © 2019 The Authors

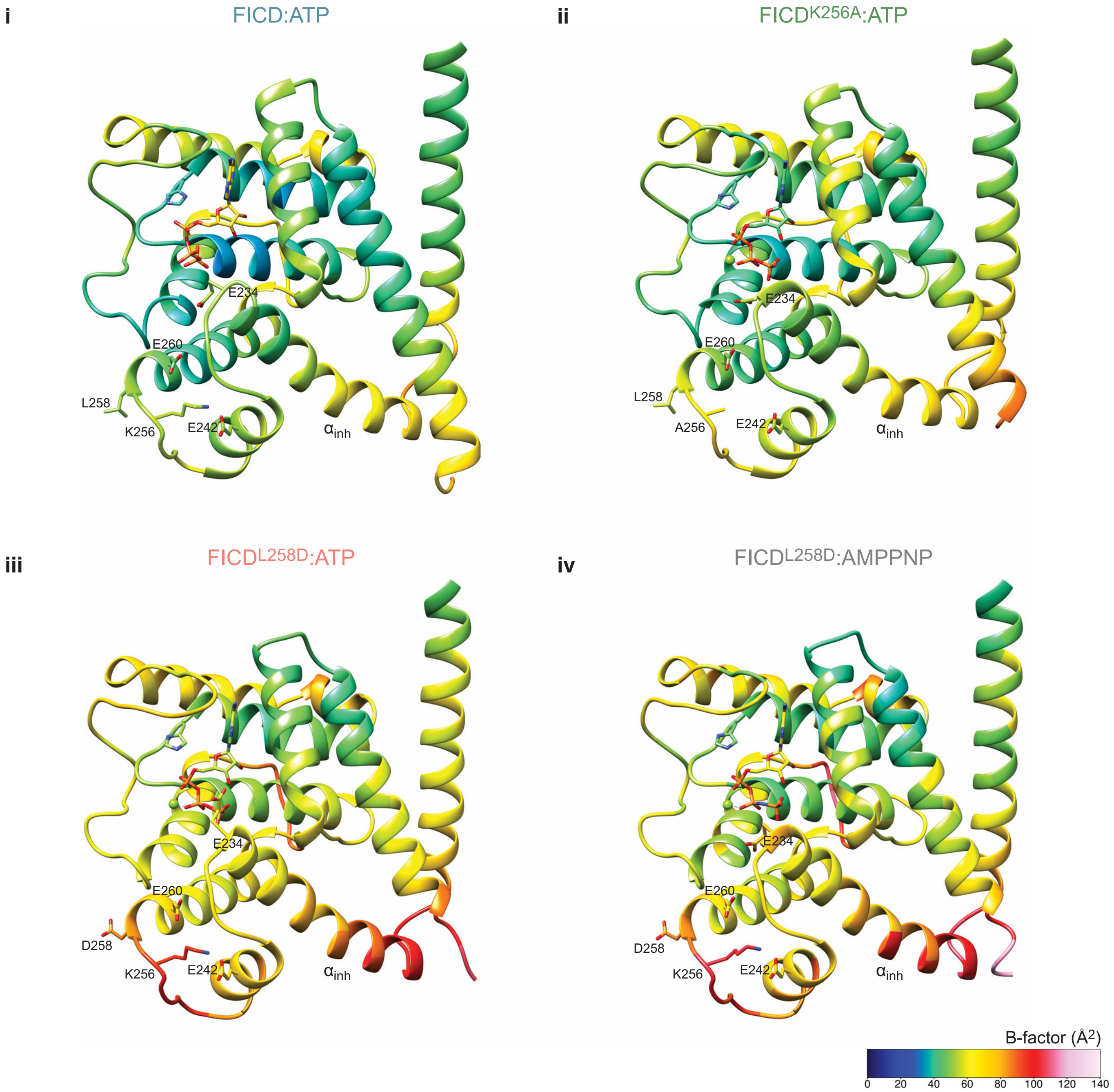

Figure EV5. AMPylation activity correlates with enhanced flexibility of the dimer interface and Glu234.

The residue average B-factors, for the four FICD complexes co-crystallised with ATP, are shown [in (i–iv)] with a cold to hot colour code. They display a trend of increasing B-factors in the dimer interface and in the inhibitory glutamate region. This increase in B-factor is indicative of increasing flexibility and correlates with greater AMPylation activity of the corresponding FICD. All of these structures have almost identical dimer packing in their respective crystals and limited crystal contacts around the inhibitory helix (see Appendix Fig S2). Note, structure averaged B-factors are comparable (see Table 1). For clarity, the TPR domain (up to residue 182) is not shown.

© 2019 The Authors

Figure EV6. ATP negatively modulates pre-AMPylation complex and FICD dimer stability.

A Immobilised BiP responds allosterically to, is saturated by and retains ATP for the duration of BLI kinetic assays. BLI traces of the interaction between $FICD^{L258D\text{-}H363A}$ and immobilised biotinylated $BiP^{T229A\text{-}V461F}$ in different nucleotide states. Before exposure to $FICD^{L258D\text{-}H363A}$ immobilised BiP:Apo was subjected to two consecutive incubation steps (activation and wash) in the presence or absence of ATP as indicated. FICD association and dissociation steps (shown) were then conducted in a nucleotide (Nt.)-free solution. Note that BiP only interacts with $FICD^{L258D\text{-}H363A}$ when pre-saturated with ATP. Importantly, ATP pre-bound BiP retains its affinity for $FICD^{L258D\text{-}H363A}$ even if subsequently washed in a buffer lacking ATP (compare red and green traces). Thus, the majority of BiP retains its bound ATP for the duration of the kinetic experiment, experimentally uncoupling the effect of nucleotide on the FICD analyte from its effect on the immobilised BiP ligand.

B Cartoon schematic of the BLI assays presented in Fig 6A and B. The pre-AMPylation complex is formed between the immobilised BiP:ATP “ligand” and the FICD “analyte”.

C The BLI association and dissociation traces from Fig 6B are shown. The immobilised biotinylated $BiP^{T229A\text{-}V461F}$ was saturated with ATP and then exposed to nucleotide-free FICDs. Dissociation was performed in absence or presence of ATP, as indicated. [$mFICD^{H363A}$: $FICD^{L258D\text{-}H363A}$; $dFICD^{H363A}$: $FICD^{H363A}$].

D Quantification of the biphasic exponential decay fitting of dissociation traces shown in Fig 6B. Relative ATP-induced changes of these kinetic parameters are given in Fig 6D. Shown are mean values $\pm$ SD from three independent experiments. Note the greater relative contribution of fast dissociation of mFICD in presence of ATP versus absence.

E Representative BLI traces of an FICD dimer dissociation experiment. The legend indicates the form of unlabelled FICD incubated with the N-terminally biotinylated FICD (at a 100-fold molar excess, prior to biosensor loading) and also the ligand present in the dissociation buffer (at 5 mM) if applicable.

F Representative dissociation data derived from (E). Probes loaded with biotinylated FICD incubated with $mFICD^{H363A}$ act as controls for non-specific association and dissociation signals, these were subtracted from the respective $dFICD^{H363A}$ traces in (E). Mono-exponential decay best-fit lines are also displayed; resulting off rates are shown in Fig 6E(ii).

▶

 © 2019 The Authors

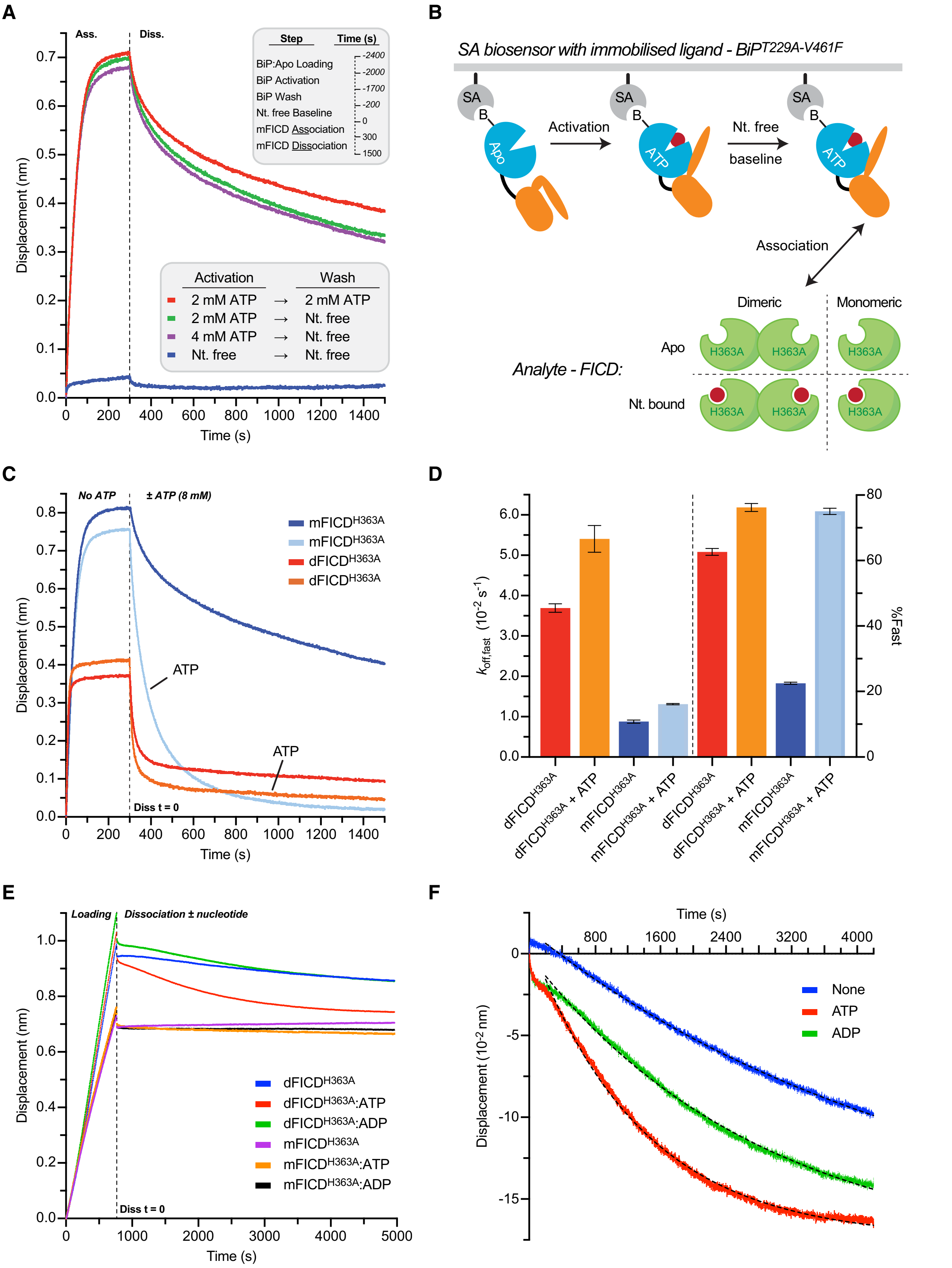

A
B
SA biosensor with immobilised ligand - BiPT229A-V461F
Activation
Nt. free baseline
Association
Dimeric
Monomeric
Apo
Nt. bound
Analyte - FICD:
C
D
E
F

Figure EV6.
