# Supplementary material for: An oligomeric state‐dependent switch in the ER enzyme FICD regulates AMPylation and deAMPylation of BiP
Source: EMBO J. 2019 Sep 18;38(21):e102177. doi: 10.15252/embj.2019102177 (PMC6826200; doi:10.15252/embj.2019102177)
Supplement: Supplementary file 3 — Table EV1 [file EMBJ-38-e102177-s003.docx]

**Table EV1**

| **Dataset** | **PDB Code** | **Crystallisation Condition *(Protein:Seeds:Well Reservoir Solution (nl))*** | **Cryoprotectant** | **Seed Protein** | **Seed Crystallisation Conditions *(Seed Dilution)*** |
| --- | --- | --- | --- | --- | --- |
| FICD:ATP | 6I7G | 0.1 M Tris pH 7.5; 20% PEG 300; 5% PEG8K; 10% Glycerol *(150:50:100)* | PFPE | FICD | 0.2 M (NH_4_)_2_SO_4_, 0.1 M NaCacodylate, 30% PEG 8000 *(1/3)* |
| FICD^K256S^:Apo | 6I7H | 0.1 M Tris pH 8.5; 0.05 M MgCl_2_; 40% EtOH  *(200:0:100)* | PFPE | N/A | N/A |
| FICD^K256A^:MgATP | 6I7I | 0.1 M Bis-Tris pH 6.5; 0.2 M MgCl_2_; 25% PEG3350 *(100:25:100)* | PFPE | FICD^K256A^ | 0.1 M Na_3_Citrate pH 5.5, 40% PEG 600 *(1/10)* |
| FICD^L258D^:Apo | 6I7J | 0.1 M Tris pH 8.5; 2.0 M (NH_4_)_2_SO_4_ *(150:50:100)* | Well solution + 30% glycerol | FICD^L258D^ | 0.1 M Tris pH 8.5, 0.2 M Li_2_SO_4_, 40% PEG 4000 *(1/2)* |
| FICD^L258D^:MgATP | 6I7K | 1.0 M NaCl; 10% EtOH *(150:50:200)* | Well solution + 30% glycerol | FICD^L258D-H363A^ | 0.1 M HEPES pH 7.5, 1 M NaOAc *(1/100)* |
| FICD^L258D^:MgAMPPNP | 6I7L | 1.5 M NaCl; 10% EtOH *(150:50:200)* | Well solution + 50% saturated sucrose | FICD^K256A^ | 0.1 M Na_3_Citrate pH 5.5, 40% PEG 600 *(1/500)* |

Crystallisation conditions and cryoprotectants used. Where applicable the crystallisation conditions (and seed dilution) of the crystals used for micro-seeding are also shown. Seed stock solutions were produced by crushing crystals formed in the relevant sitting drop and resuspending in the respective reservoir solution (ca. 70 µl). Note, PEG percentage is given in w/v and EtOH percentage in v/v.
