# Supplementary figures and images for: An oligomeric state‐dependent switch in the ER enzyme FICD regulates AMPylation and deAMPylation of BiP
Source: EMBO J. 2019 Sep 18;38(21):e102177. doi: 10.15252/embj.2019102177 (PMC6826200; doi:10.15252/embj.2019102177)

Appendix Figure 1C

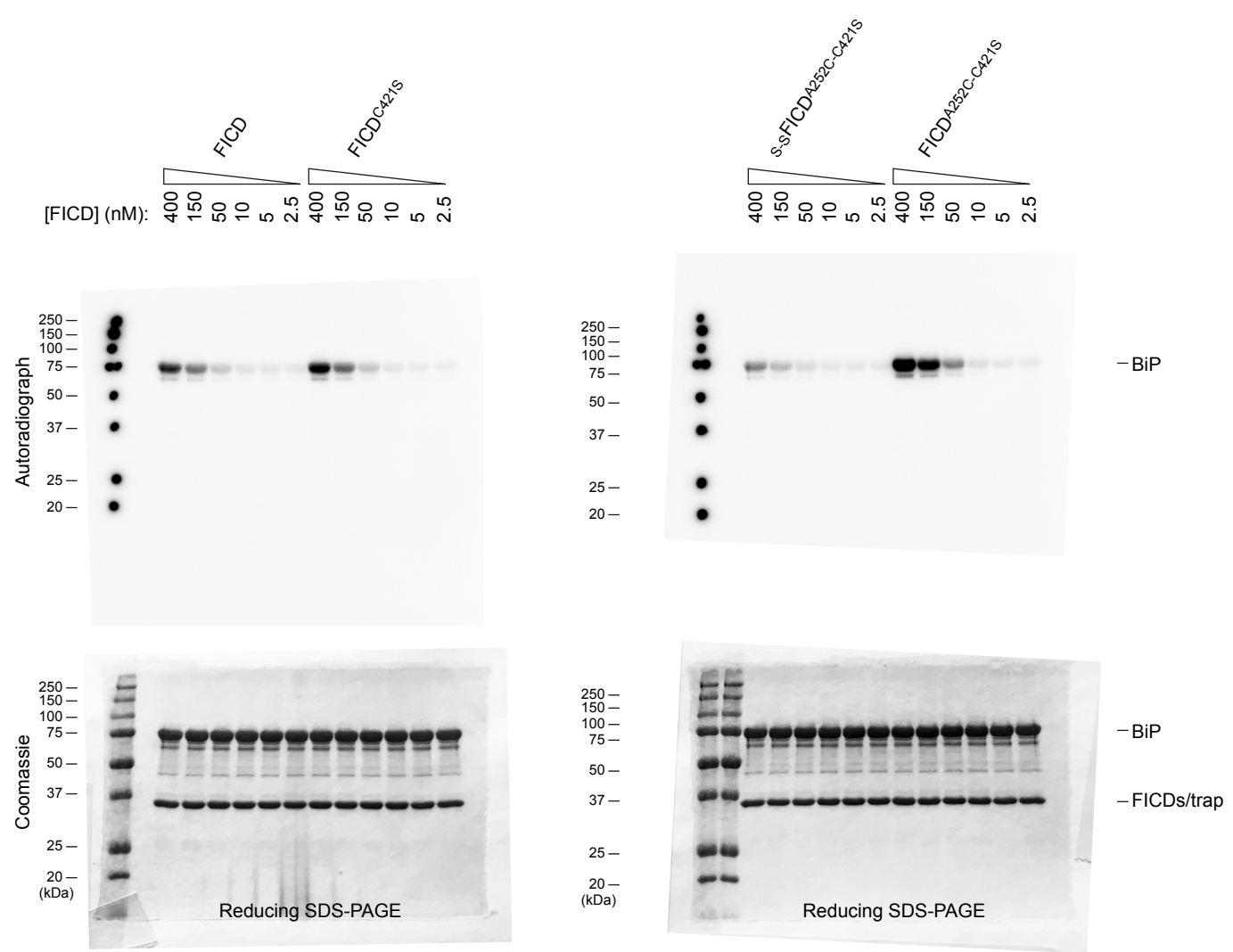

Supplement: Supplementary file 5 — Source Data for Expanded View and Appendix [file EMBJ-38-e102177-s010.zip › Appendix_Figure1C_uncropped.pdf]

EV3A

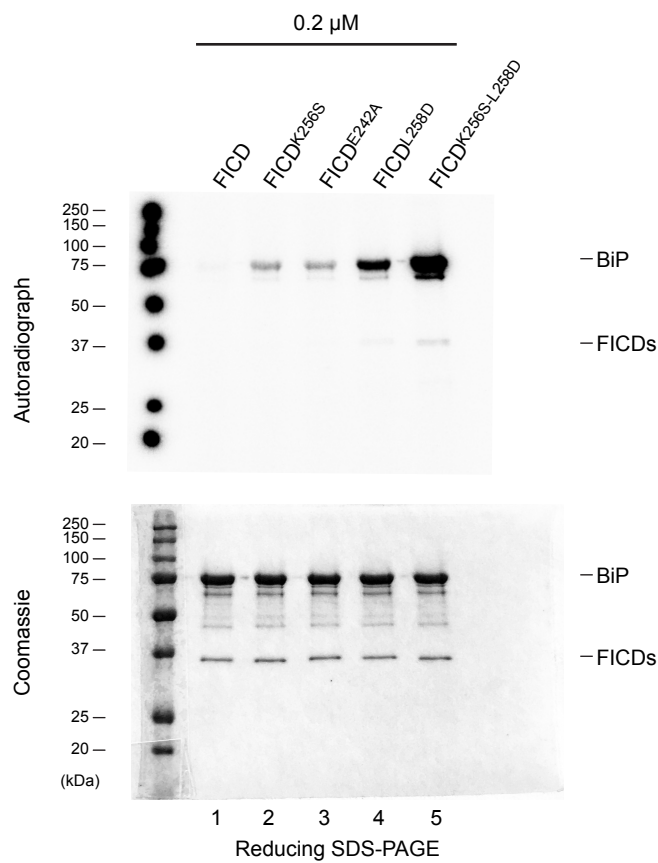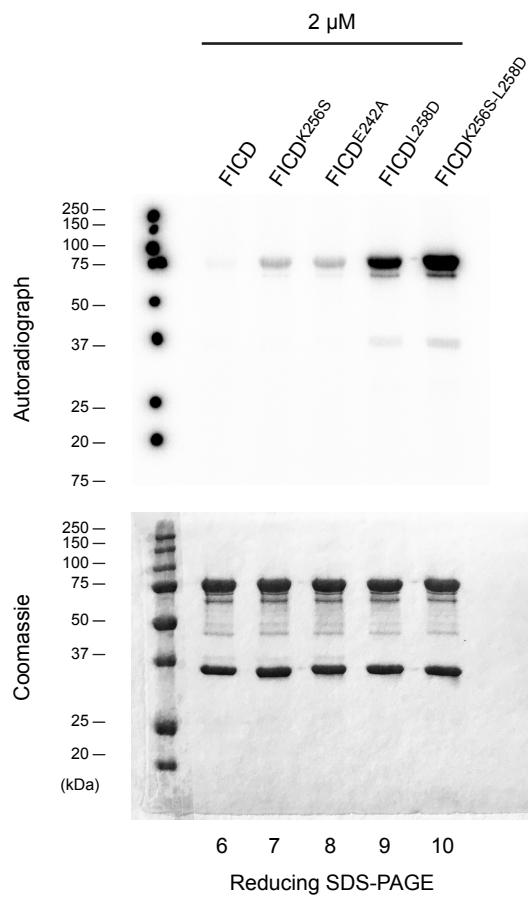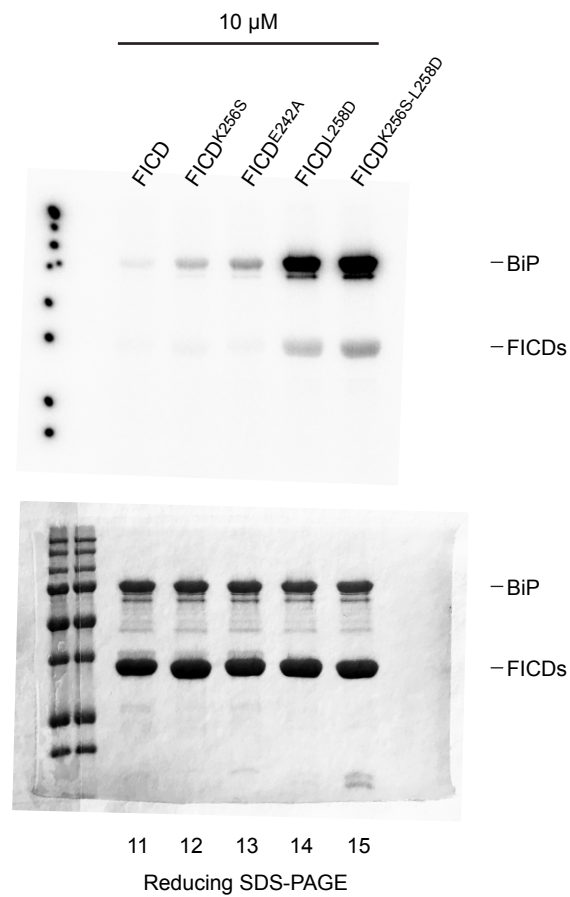

Supplement: Supplementary file 5 — Source Data for Expanded View and Appendix [file EMBJ-38-e102177-s010.zip › EV3A_uncropped.pdf]

Figure 1A

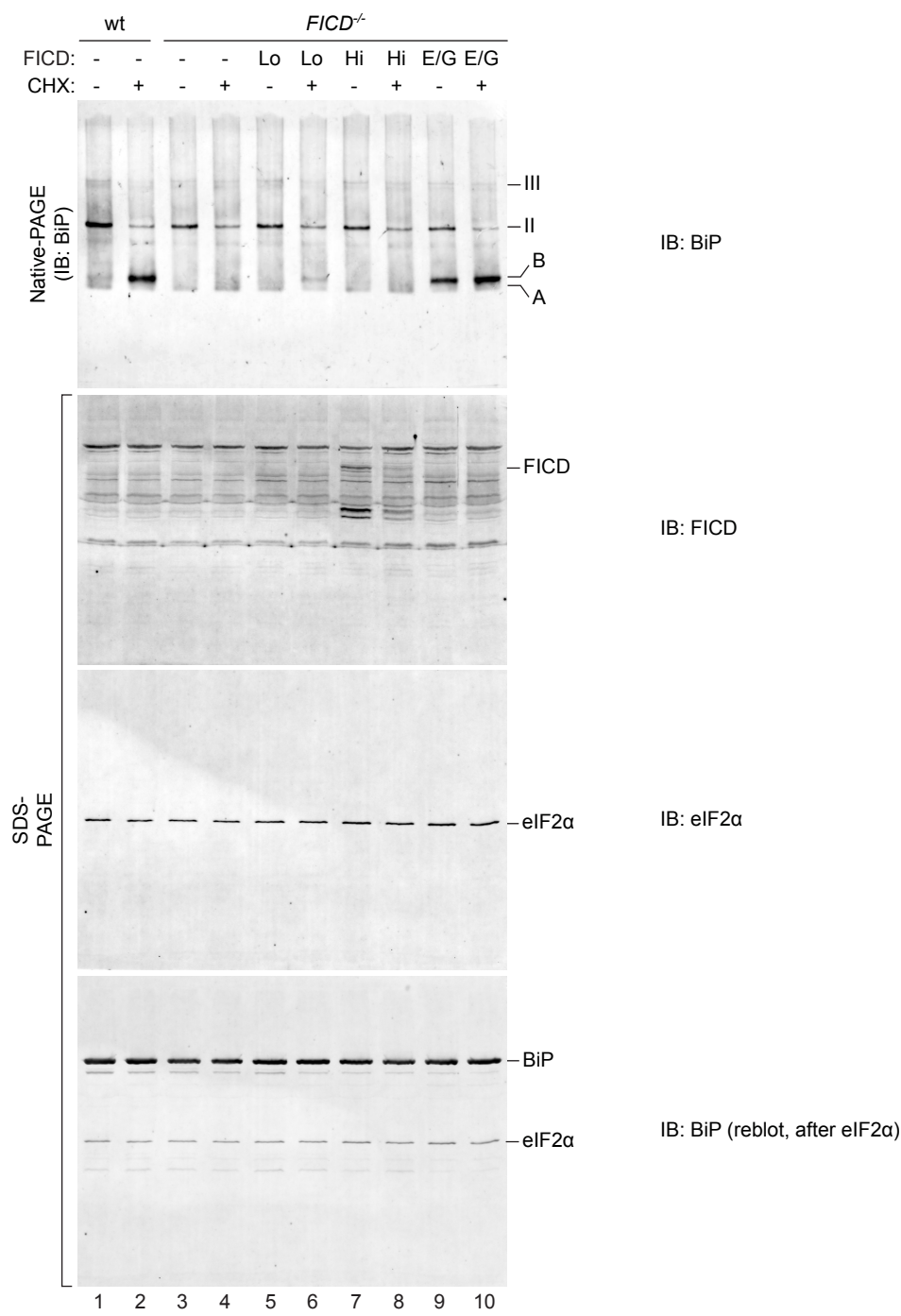

Figure 1B

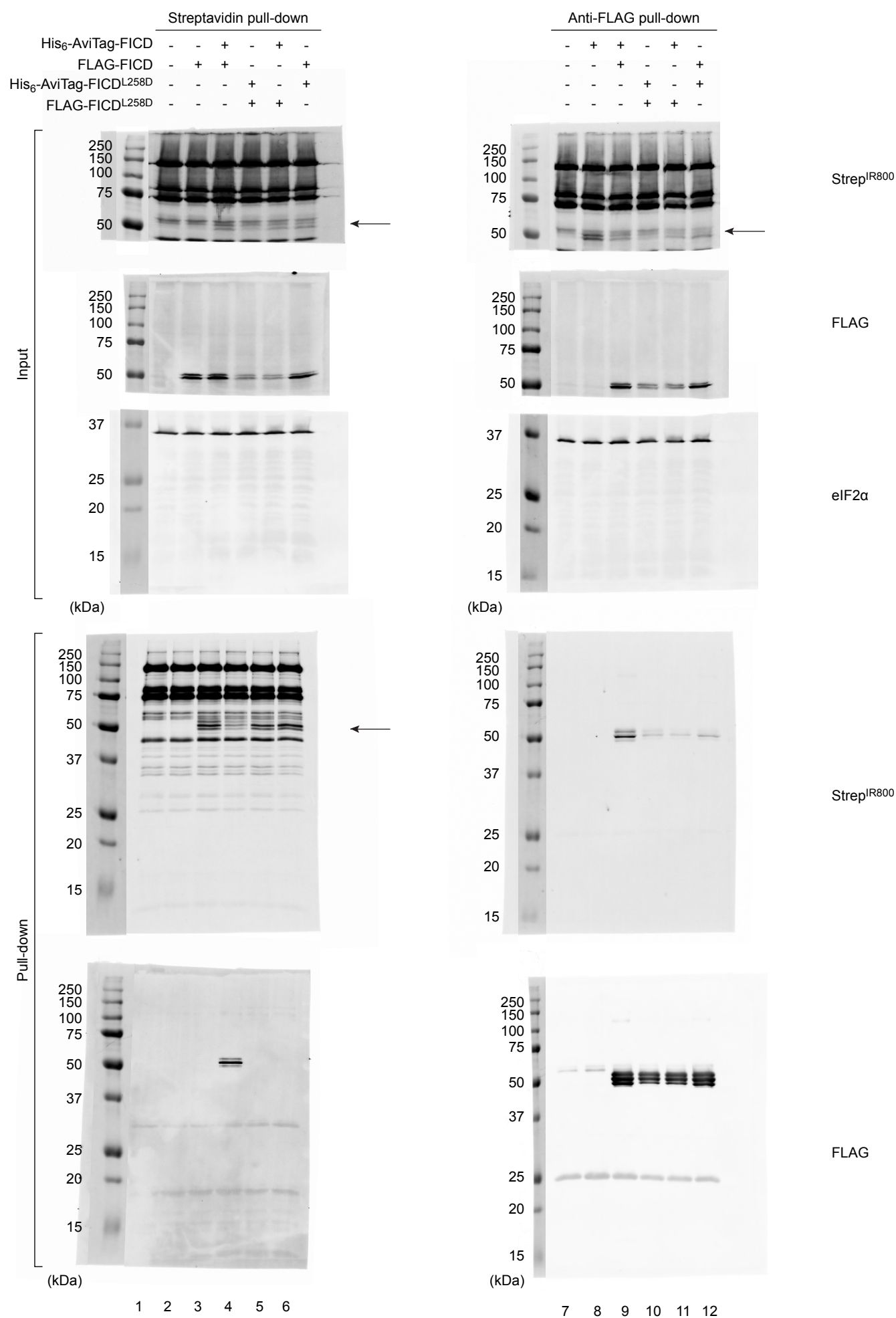

Figure 1C

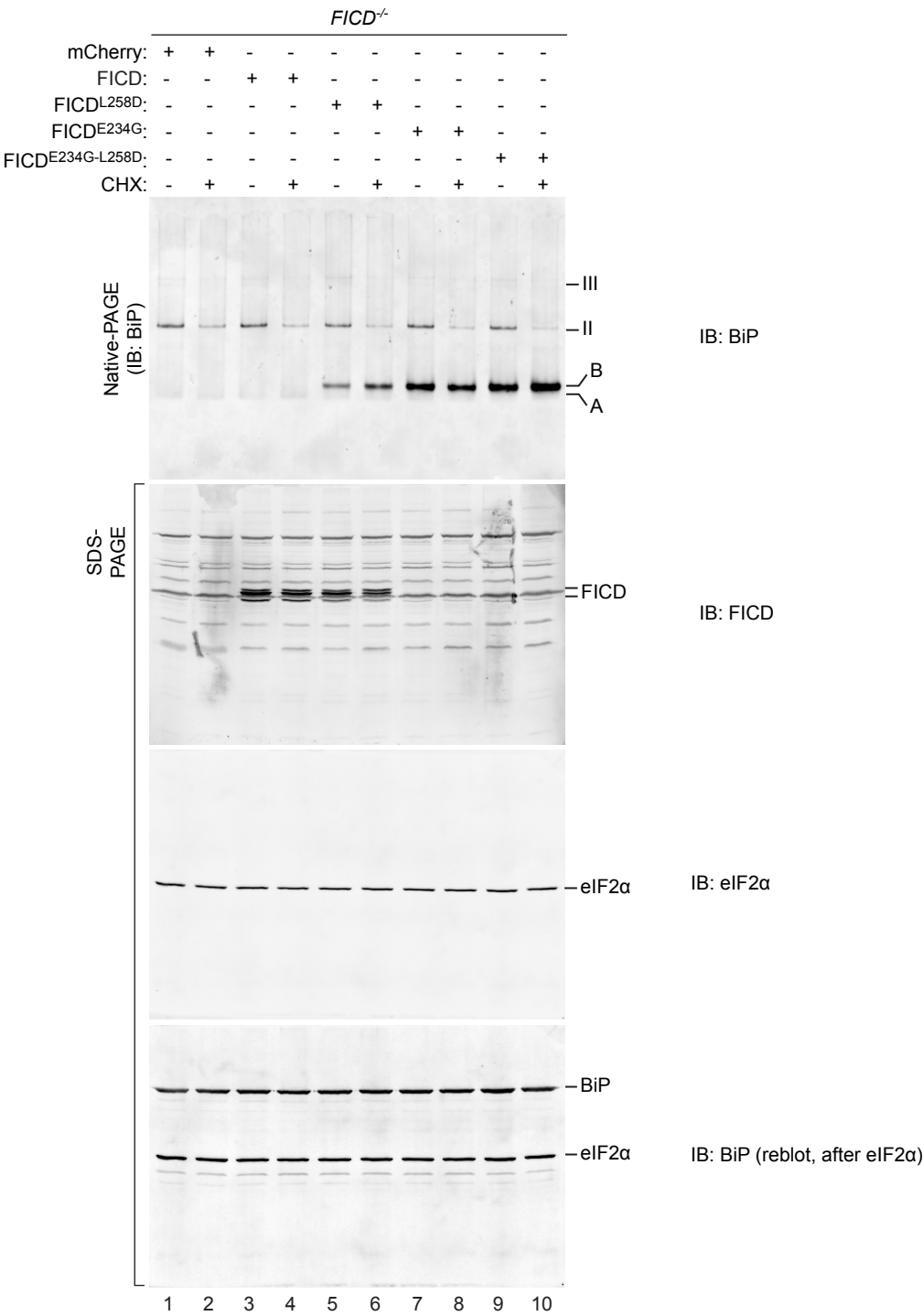

Figure 1F

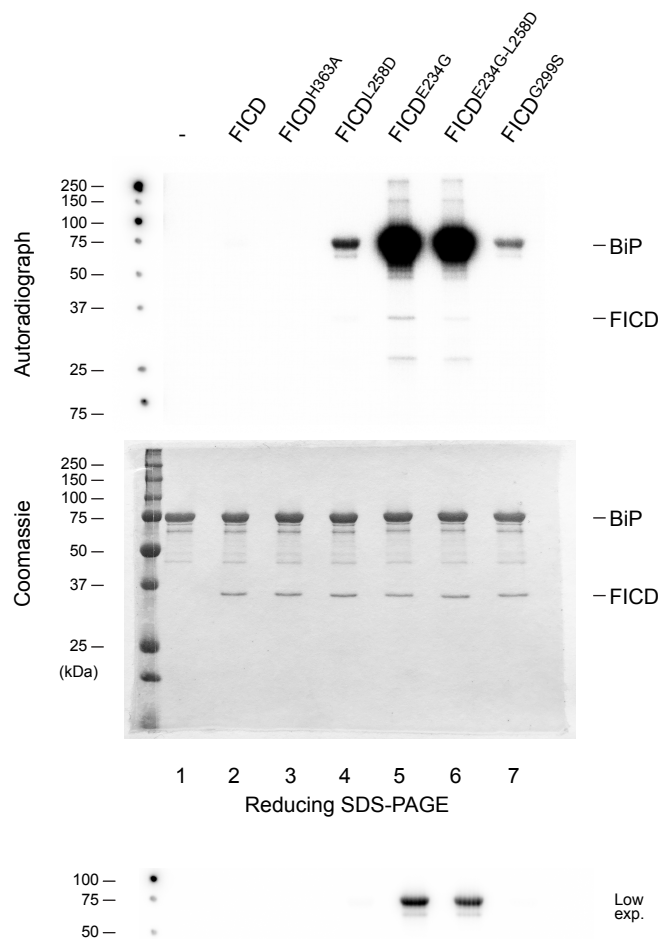

Supplement: Supplementary file 7 — Source Data for Figure 1 [file EMBJ-38-e102177-s005.zip › Figure_1_Source_Data.pdf]

Figure 2E

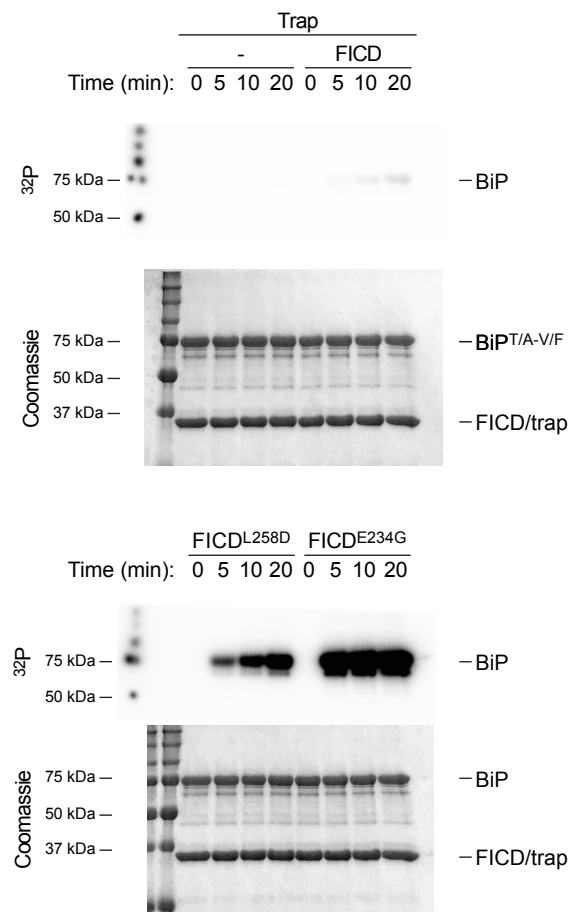

Supplement: Supplementary file 8 — Source Data for Figure 2 [file EMBJ-38-e102177-s006.zip › Figure_2E_uncropped_images.pdf]

Figure 3A

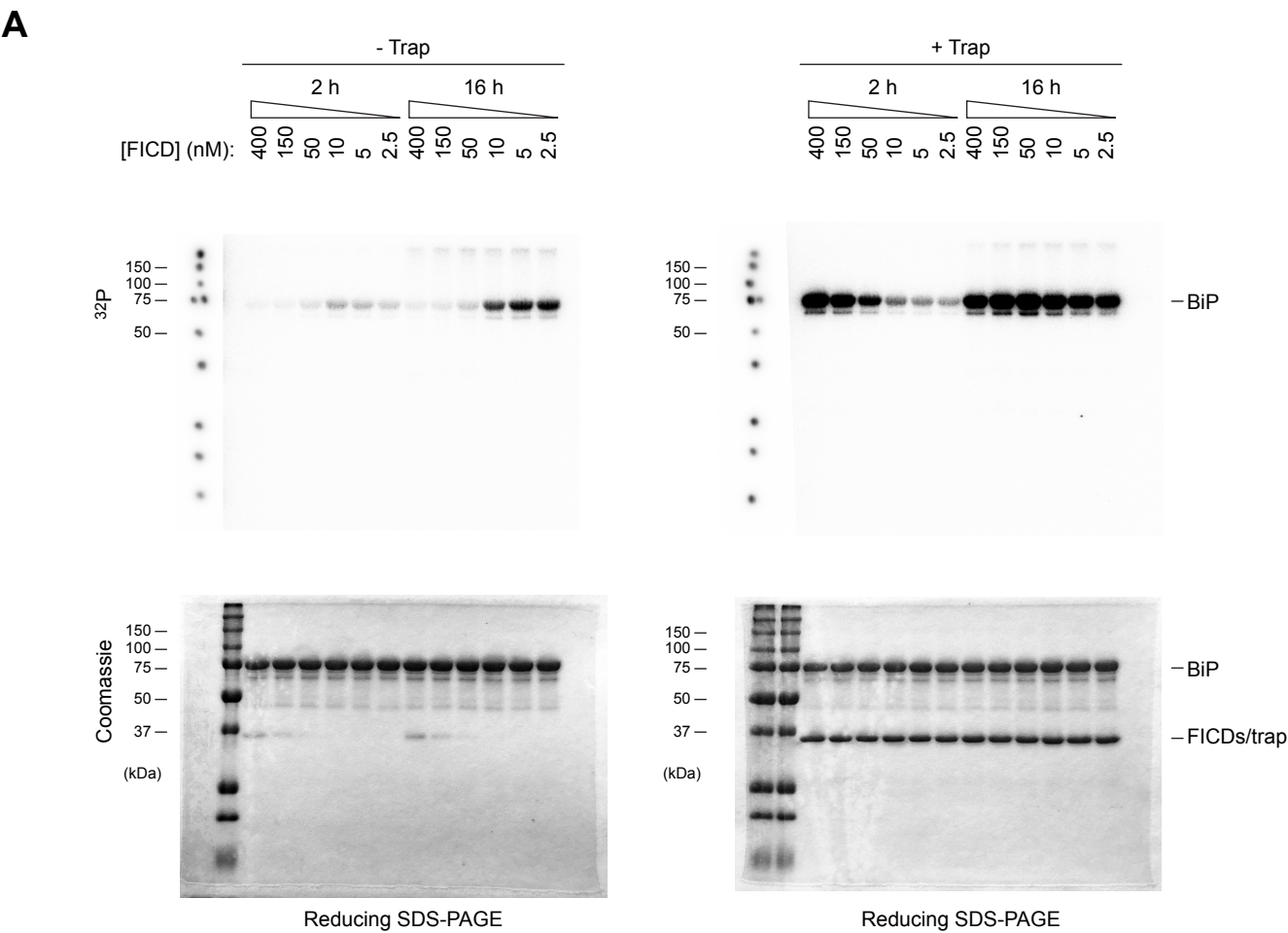

Figure 3B

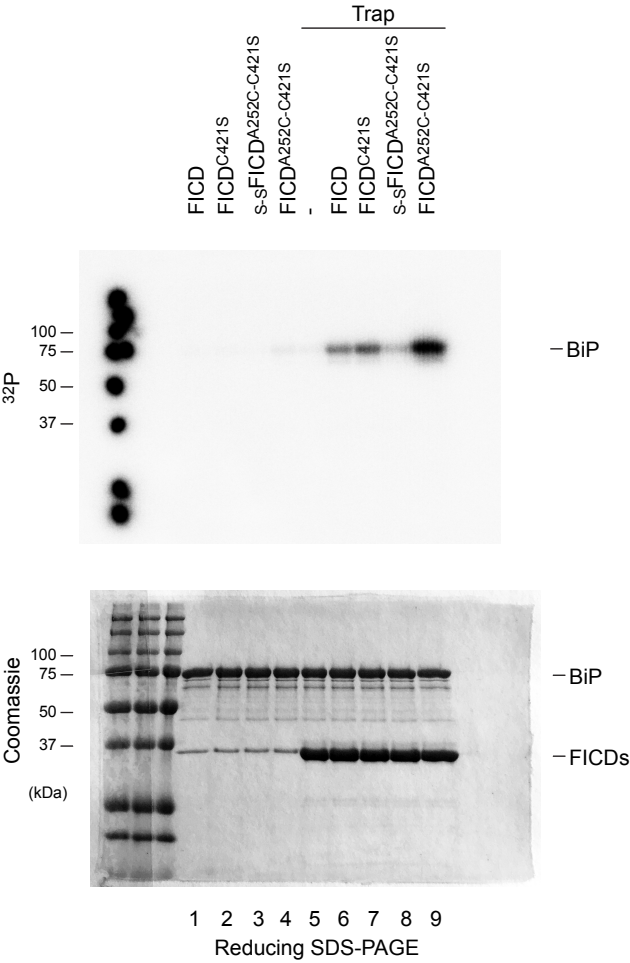

Figure 3C

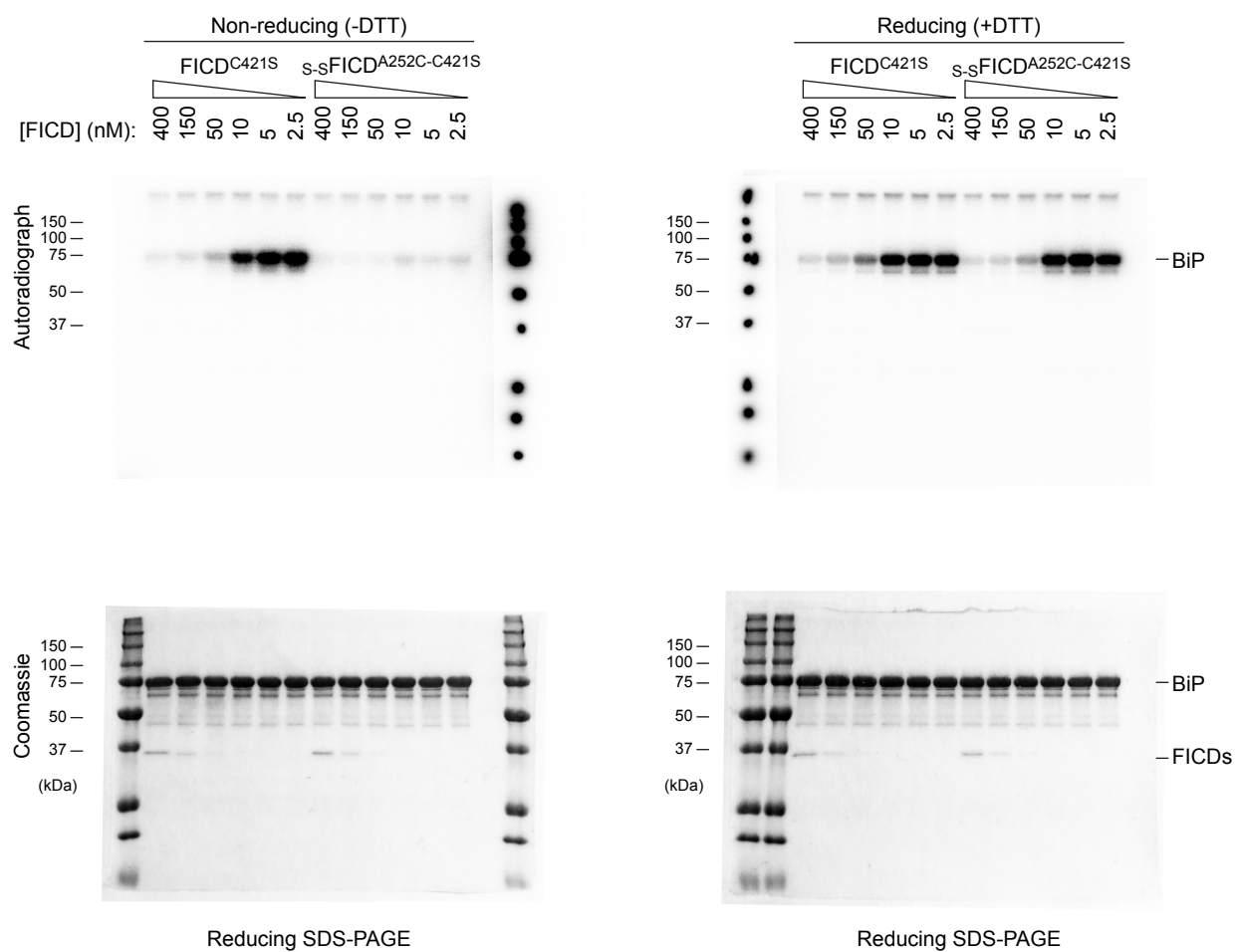

—●— S-S FICD<sup>A252C-C421S</sup>

Supplement: Supplementary file 9 — Source Data for Figure 3 [file EMBJ-38-e102177-s007.zip › Figure_3_Source_Data.pdf]

Figure 4C

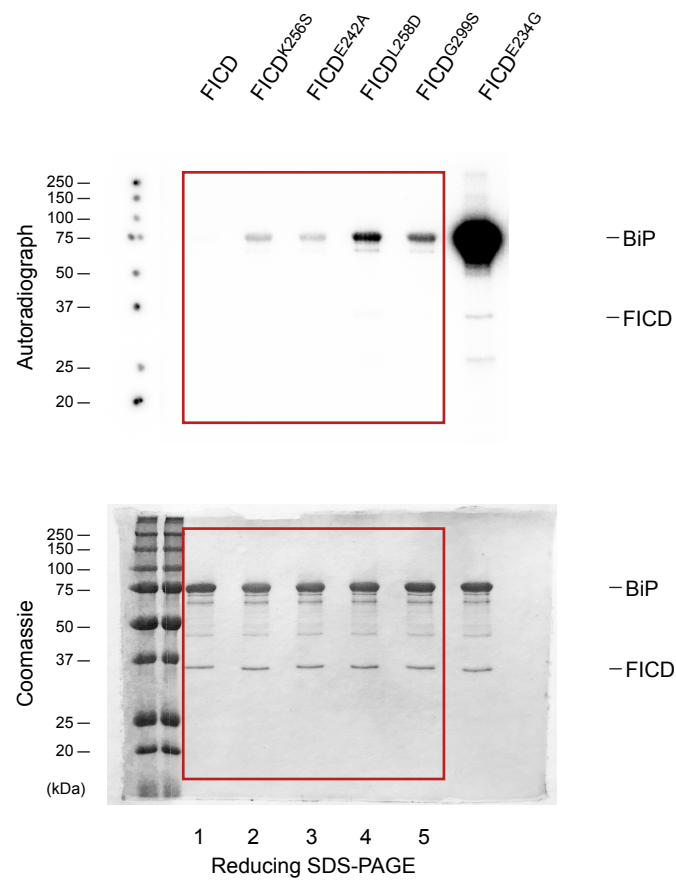

Supplement: Supplementary file 10 — Source Data for Figure 4 [file EMBJ-38-e102177-s008.zip › Figure_4C_uncropped_images.pdf]
